# Aβ Peptide Fibrillar Architectures Controlled by Conformational Constraints of the Monomer

**DOI:** 10.1371/journal.pone.0025157

**Published:** 2011-09-29

**Authors:** Kristoffer Brännström, Anders Öhman, Anders Olofsson

**Affiliations:** 1 Department of Medical Biochemistry and Biophysics, Umeå University, Umeå, Sweden; 2 Department of Chemistry, Umeå University, Umeå, Sweden; University of Arkansas for Medical Sciences, United States of America

## Abstract

Anomalous self-assembly of the Aβ peptide into fibrillar amyloid deposits is strongly correlated with the development of Alzheimer's disease. Aβ fibril extension follows a template guided “dock and lock” mechanism where polymerisation is catalysed by the fibrillar ends. Using surface plasmon resonance (SPR) and quenched hydrogen-deuterium exchange NMR (H/D-exchange NMR), we have analysed the fibrillar structure and polymerisation properties of both the highly aggregation prone Aβ1–40 Glu22Gly (Aβ^40Arc^) and wild type Aβ1–40 (Aβ^40WT^). The solvent protection patterns from H/D exchange experiments suggest very similar structures of the fibrillar forms. However, through cross-seeding experiments monitored by SPR, we found that the monomeric form of Aβ^40WT^ is significantly impaired to acquire the fibrillar architecture of Aβ^40Arc^. A detailed characterisation demonstrated that Aβ^40WT^ has a restricted ability to dock and isomerise with high binding affinity onto Aβ^40Arc^ fibrils. These results have general implications for the process of fibril assembly, where the rate of polymerisation, and consequently the architecture of the formed fibrils, is restricted by conformational constraints of the monomers. Interestingly, we also found that the kinetic rate of fibril formation rather than the thermodynamically lowest energy state determines the overall fibrillar structure.

## Introduction

Alzheimer's disease (AD) is the most common form of dementia today and results in both individual suffering and major economical costs for society [1]. Development of AD is strongly correlated to aggregation and amyloid formation of the 38–43 residue long amyloid-β peptide (Aβ). Aβ peptide is derived via sequential cleavages of the amyloid precursor protein (APP) by β- and γ-secretases and is a natively unfolded peptide having a high propensity to aggregate into cross-β amyloid fibrils through a nucleation-dependent mechanism. The reason why Aβ peptides detrimentally self-assemble into fibrils in certain individuals is currently not well understood. In most AD cases, no underlying factor for the development of the disease can be pinpointed. However, within a small group of individuals with early onset Alzheimer's disease (EOAD), a Mendelian inheritance has been observed [2]. For most of these cases, a genetic anomaly results in an enhanced processing of APP followed by increased Aβ levels, ultimately resulting in an increased rate of aggregation and fibril formation [3]. In a few cases of EOAD, the phenotype has been linked to a mutation within the Aβ sequence of the APP gene resulting in a gain of function where increased aggregation is observed. The Aβ Glu22Gly mutation was identified in northern Sweden and is frequently denoted as the Arctic Aβ variant, Aβ^40Arc^. This mutation is associated with an aggressive form of AD [4], [5], [6]. Aβ Glu22Gly has a very high propensity for aggregation and an enhanced ability to form protofibrils that have been shown to be cytotoxic in both cell culture and transgenic animals [5], [7], [8], [9], [10], [11], [12], [13].

Amyloid polymerisation is a complicated process where monomers are incorporated into the amyloid fibril form in several steps. The polymerisation process has been studied in detail and it is widely accepted that the process involves a dock and lock mechanism [14]. The accepted model involves an initial docking step that is followed by an affinity maturation reaction through an isomerisation phase resulting in adjustment into a conformation with higher binding strength. The model has been further developed into 3 steps based on a dock, lock and block mechanism where an initial weak association is followed by a time dependent maturation phase with a concomitant increase in binding affinity [15]. As each subunit is incorporated, a novel recognition site is created and subsequent binding of additional peptides blocks dissociation. This polymerisation phenomenon can be conveniently monitored using surface plasmon resonance (SPR) where pre-formed fibrils are immobilised and polymerisation is monitored through the addition of monomeric Aβ peptides [15], [16], [17], [18].

Within this work we can show that the architecture of Aβ fibrils is determined by constraints imposed by the monomer conformation during docking and isomerisation. This finding has general implications and suggests that although Aβ is considered to be unstructured it is restrained to adopt certain conformations which impair its ability to acquire certain fibrillar structures. Interestingly, our results moreover show that the kinetic rate of fibril formation determines the fibrillar architecture rather than the thermodynamically lowest energy state.

## Materials and Methods

### Preparation of monomeric peptide and fibrillar samples

All peptides were obtained from Alexotech AB, Umeå, Sweden (www.alexotech.com). Due to the aggregation properties of Aβ-peptides, an appropriate solubilisation scheme was essential. Prior to use, lyophilised peptides were solubilised in 10 mM NaOH at pH 12, followed by 30 s sonication in a water bath and 5 min centrifugation at 20 000 g to remove residual oligomeric species. This treatment efficiently monomerised the peptides and facilitated dilution in phosphate buffered saline (PBS, 15 mM phosphate buffer, pH 7.4, 150 mM NaCl) to the selected concentrations. All samples were verified to have pH 7.4 or a pD corresponding to 7.0 by direct pH meter reading. Fibrillar forms were acquired by incubating 100 µM Aβ at 37°C under agitation for 48 hours. μ.

### H/D exchange and NMR analysis of Aβ^40WT^ and Aβ^40Arc^ fibrils

The fibrillar forms of ^15^N-Aβ^40WT^ and ^15^N-Aβ^40Arc^ were prepared as described above. Fibrils composed of ^15^N-Aβ^40Arc^ were also prepared by cross-seeding using 15% (w/w) ^14^N-Aβ^WT40^ fibrils as seeds. Agitation was omitted during cross-seeding experiments.

To probe solvent accessibility, aggregate/fibrillar solutions of each peptide type were split into two fractions and recovered by short centrifugation at 20 000 g. Hydrogen-deuterium (H/D) exchange was carried out on one of the fractions by diluting the pellets 30 times using a D_2_O solution (20 mM Tris, 150 mM NaCl, pD 7.0) followed by 24 h incubation at 37°C. The second fraction was used without further treatment as a fully protonated reference sample to identify and exclude amide exchange resulting from the experimental procedure (i.e. highly exposed amides within the monomeric state). At the end of the incubation period and immediately prior to NMR analysis, Aβ assemblies were recovered by short centrifugations (20 000 g) and rapidly converted into NMR-detectable monomers using an optimised solution of hexafluoroisopropanol as described previously [19].

Hydrogen exchange was subsequently monitored by recording a series of heteronuclear 2D ^15^N-HSQC experiments, typically starting 6–8 minutes after fibril dissolution. All experiments were performed at 15°C on a 600 MHz Bruker AVANCE spectrometer, equipped with a 5 mm triple-resonance, pulsed-field z-gradient cryoprobe. The acquisition time for each ^15^N-HSQC experiment was 10 min using four transients per increment and 128 (t_1_)×1024 (t_2_) complex data points. Prior to each ^15^N-HSQC experiment, a 1D proton NMR spectrum was acquired to quantitatively monitor the dissolution of fibrils into monomers. Protection ratios and experimental errors were determined as described previously [19], [20], [21], [22], [23], [24], [25].

### Surface plasmon resonance (SPR)

The different Aβ variants, including both monomeric and fibrillar, forms were immobilized at 10 µM peptide concentration to a final density corresponding to 2000–5 000 RU to a CM5 chip or to the dextran free chip C1 (GE Healthcare) using standard amine-coupling chemistry at pH 5. Briefly, to activate the chip a 50/50 mixture of EDC (*N*-(3-dimethylaminopropyl)-*N*′-ethylcarbodimidehydrochloride) 0.4 M and NHS (*N*-hydroxysuccinimide) 0,1 M for 7 min.

After immobilization the chip was inactivated with a 7 min injection of etanolamine 1 M.

Analysis of monomeric Aβ binding to fibrils was performed at a flow rate of 20 µl/min in PBS at 25°C. Important to note is that the SPR signal is affected by the physical distance of the analyzed interaction and the chip-surface. Due to the intrinsic nature of a polymerising reaction the distance between the fibrillar ends and the surface will increase during the reaction. Upon extended polymerisation this results in a decreasing SPR signal and a non-linear response. This effect was in detail monitored and to avoid the problem of a non-linear dependency, fibrillar extension was kept at a minimum, using short injection times in combination with low peptide concentration, well within the range where a non-linear curvature becomes pronounced.

According to standard procedures all sensograms were corrected for non-specific interactions to a reference surface, and by double referencing [26]. Regarding a subsequent analysis of the fibril morphology Aβ fibrils were immobilized to 1000RU on a C1 chip followed by injection of monomeric Aβ allowing the fibrils to grow to at least 3000RU. The SPR chip was then dissembled and directly analyzed using AFM as described below.

### Affinity determination between free peptide and the amyloid fiber using SPR

Measurements of binding affinities between free monomers and an amyloid fibril is not straight forwards since saturation of monomer binding cannot be reached. However, since the concentration of fibrils is constant during polymerisation, the monomer dissociation constant will be equal to the free concentration (critical concentration) of monomers at equilibrium. Therefore, the dissociation constant can be used in combination with SPR data obtained with known concentrations of peptides in the running buffer to determine the affinity between the monomers and fibrillar ends [27], [28]. Fibril extension of Aβ was initiated through injection of 2 µM onto preformed immobilized fibrils at a flow rate of 20 µl/min in PBS at 25°C for in total 30 s followed immediately by various injection of 0–400 nM Aβ solution using the feature COINJECTION. The specific concentration of Aβ in the second injection that produced a linear plateau response (no dissociation observed) represent the critical concentration for polymerisation and consequently also the binding constant.

### Atomic force microscopy (AFM) analysis

Fibrillar samples were analysed directly on the surface of C1 chips (GE Healthcare, Uppsala, Sweden) that do not have a dextran surface. Analysis was performed using a Nanoscope IIIa multimode AFM (Digital Instruments Santa Barbara, USA) in tapping mode™ in air. A silicon probe was oscillated at around 280 kHz and images were collected at an optimised scan rate corresponding to 1–2 Hz.

## Results

### Fibrillar core analysis using quenched H/D exchange NMR

**Figure 1:**
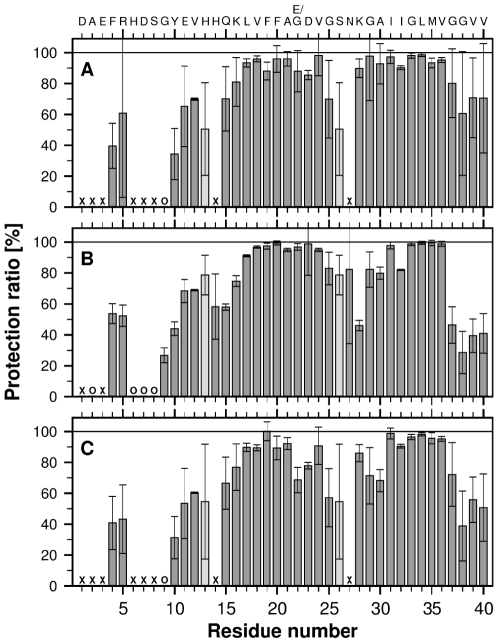
Solvent protection ratios for backbone amide protons as determined by quenched H/D exchange monitored by NMR spectroscopy. Protection is defined as the ratio of the observed signal intensity after a 24 h pre-incubation period in D_2_O to the signal intensity in a completely protonated reference sample. Protection in the reference sample is defined as 100%. Circles correspond to residues with 0% protection and crosses to residues where exchange was too fast for detection. Pale grey bars indicate overlapping residues with ambiguously assigned protection ratios. Error bars indicate the experimental uncertainty given by the measurements. (A) Aβ^40Arc^ fibrils, (B) Aβ^40WT^ fibrils, and (C) Aβ^40Arc^ seeded with Aβ^40WT^ fibrils.

To evaluate whether there are any structural differences between the fibrillar cores of Aβ^40Arc^ and Aβ^40WT^, their corresponding fibrillar form were analysed through quenched H/D exchange NMR experiments. Figure 1a and 1b illustrates the solvent protection of the core structure of Aβ^40Arc^ and Aβ^40WT^, respectively. The result clearly shows that no significant difference can be identified between the two forms, only a slightly lower protection is observed for the four C-terminal residues of Aβ^40WT^. Figure 1c illustrates the H/D exchange pattern acquired from Aβ^40Arc^ when seeded with Aβ^40WT^ fibrils and further strengthen the notion that no obvious difference can be detected on the backbone structure, apart from a moderate reduction of the solvent protection at the mutation site of Aβ^40Arc^.

### Fibril polymerisation of Aβ^40WT^ and Aβ^40Arc^ monitored by SPR

**Figure 2:**
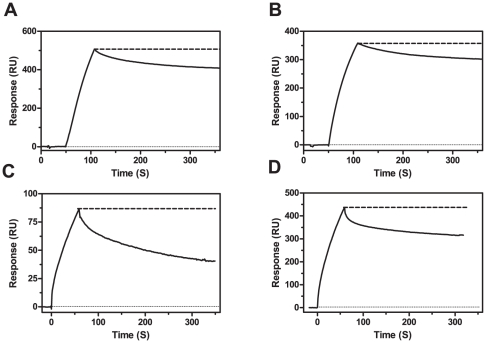
SPR study of fibril elongation. Pre-formed Aβ fibrils were immobilised on a CM5 chip and probed with 2 µM monomeric Aβ for 1 min at a flow rate of 20 µl/min in PBS at 25°C. (A) Monomeric Aβ^40WT^ seeded with Aβ^40WT^ fibrils, (B) monomeric Aβ^40Arc^ seeded with Aβ^40Arc^ fibrils, (C) monomeric Aβ^40WT^ seeded with Aβ^40Arc^ fibrils, and (D) monomeric Aβ^40Arc^ seeded with Aβ^40WT^ fibrils.

Surface plasmon resonance (SPR) enables the changes in bound mass to a surface to be monitored and therefore provides a convenient tool to follow fibril formation. Immobilised Aβ^40WT^ fibrils recruited free Aβ^40WT^ monomers by a fibrillar polymerisation process (Fig. 2a). Binding sensograms hence displayed an association phase immediately upon injection of monomeric Aβ peptides as a result of a continuous polymerisation, followed by a dissociation phase at the end of each injection. The same result was observed for immobilised fibrils of Aβ^40Arc^ and free Aβ^40Arc^ monomers (Fig. 2b). To further examine the intermolecular interactions between Aβ^40WT^ and Aβ^40Arc^, cross-seeding experiments were also performed. Interestingly, only a very slow polymerisation was observed upon exposing monomeric Aβ^40WT^ to Aβ^40Arc^ fibrils accompanied by a more rapid dissociation phase (Fig. 2c). In contrast, Aβ^40Arc^ monomers were easily recruited by Aβ^40WT^ fibrils and not accompanied with an increase in dissociation rates (Fig. 2d). The effects on polymerisation and dissociation rates for Aβ^40WT^ monomers persisted even after cross-seeding with Aβ^40Arc^/Aβ^WT40^ fibrils suggesting that it is the fibrillar architecture rather than a sequence specific effect that is causing this effect (data not shown). As a consequence, two different fibrillar architectures can be anticipated where Aβ^40Arc^ monomers can more easily polymerise onto either fibrillar form than Aβ^40WT^ monomers.

Immobilised monomeric Aβ on the chip surface could not recruit monomeric variants from the solution, probed in an identical manner as described above. This important control verified the specificity of the system and also highlights the dependency of a specific structure for an efficient peptide assembly (data not shown).

### Ultra-structure of Aβ^40WT^ and Aβ^40Arc^ fibrils

**Figure 3:**
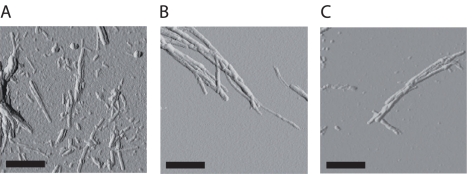
AFM analysis of fibrils immobilised on a C1 chip. Mature fibrils were briefly sonicated prior to immobilisation on C1 chip followed by continuous polymerisation with free monomers until the total mass doubled. (A) Monomeric Aβ^40WT^ seeded with Aβ^40WT^ fibrils, (B) monomeric Aβ^40Arc^ seeded with Aβ^40Arc^ fibrils, and (C) monomeric Aβ^40Arc^ seeded with Aβ^40WT^ fibrils. Scale bar is 0.5 µm.

An SPR chip without dextran (C1, GE Healthcare, Uppsala Sweden) was employed to compare Aβ^40WT^ and Aβ^40Arc^ fibril morphologies and verify preservation of fibril integrity during immobilisation on the SPR chip. Sonicated fibrils of Aβ^40WT^ or Aβ^40Arc^ were immobilised followed by a continuous polymerisation. The surface of the C1 chips were directly analysed using AFM (Fig. 3). The predominant fibrillar morphology had a diameter of 5 nm but all samples also contained thinner filaments of 3 nm diameter. No significant differences in morphology between the different samples were observed.

### Aβ^40Arc^ displays stronger binding to fibrils than Aβ^40WT^

**Figure 4:**
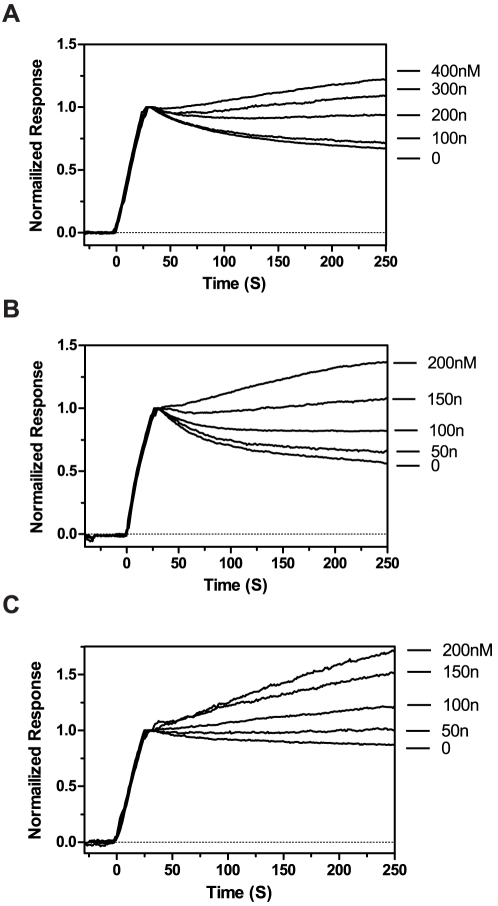
Determination of the critical free concentration of Aβ required for fibril polymerisation. 2 µM Aβ solution was injected for 30 s at a flow rate of 20 µl/min in PBS at 25°C. 100 µl of Aβ (0–400 nM) was then immediately injected. Measurements were carried out to determine the critical free concentrations for (A) Aβ^40WT^ monomers with Aβ^40WT^ fibrils, (B) Aβ^40Arc^ monomers with Aβ^40Arc^ fibrils, and (C) Aβ^40Arc^ monomers with Aβ^40WT^ fibrils.

The critical concentration of free monomers at equilibrium was determined by SPR using a co-injection technique where the dissociation phase is monitored and modulated after monomer injection by varying the concentrations of monomer in the running buffer during the decay phase [29]. The KD50 for monomeric Aβ^40WT^ and Aβ^40WT^ fibrils was determined to be 200 nM (Fig. 4a). This result is consistent with a previous report [29]. The KD50 for monomeric Aβ^40Arc^ and Aβ^40Arc^ fibrils was slightly higher (100 nM, Fig. 4b). Surprisingly, binding of monomeric Aβ^40Arc^ to Aβ^40WT^ fibrils had the strongest interaction with a KD50 value of 50 nM (Fig. 4c).

### Docking and isomerisation of Aβ^40Arc^ is enhanced

**Figure 5:**
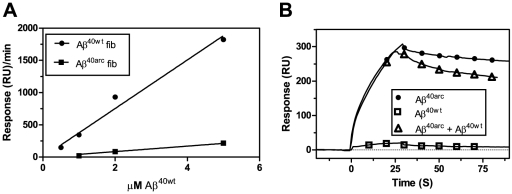
Competition between Aβ^40WT^ and Aβ^40Arc^ for polymerisation with Aβ^40Arc^ or Aβ^40WT^ fibrils. (A) Dose response for Aβ^40WT^ binding to Aβ^40Arc^ fibrils (filled squares) or Aβ^40WT^ fibrils (filled circles). A range of concentrations of Aβ^40WT^ were injected over Aβ^40Arc^ or Aβ^40WT^ fibrils in PBS at 25°C. The maximum response at the end of each injection was plotted against the concentration of Aβ^40WT^. (B) Competition between Aβ^40WT^ and Aβ^40Arc^ for polymerisation to Aβ^40Arc^ fibrils. Aβ^40WT^ was injected for 30 s at a flow rate of 20 µl/min in PBS at 25°C. 5 µM Aβ^40Arc^ (filled circles), 5 µM Aβ^40Arc^+10 µM Aβ^40WT^ (open triangles), 10 µM Aβ^40WT^ (open squares).

The finding of an impaired ability for of Aβ^40WT^ to adopt the fibrillar architecture of Aβ^40Arc^ fibrils implies an energetic barrier. According to the principle of a template- dependent dock and lock mechanisms, the locking of a peptide cannot efficiently occur unless the previously loaded peptide has assembled into the correct position [14], [15]. A scenario where locking of the peptide (i.e. affinity maturation), is the rate limiting step would be pronounced at higher peptide concentrations and decrease at lower concentrations peptide concentrations. The concentration dependence of fibril polymerisation was hence investigated to determine if peptide locking is a rate limiting step. A clear difference in association rates was observed when different concentrations of monomeric Aβ^40WT^ were polymerised with Aβ^40Arc^ or Aβ^40WT^ fibrils (Fig. 5a). However, the relative difference in association rate was not reduced at lower concentrations upon comparing the two different systems. This can be seen as a linear dependence of the association rate versus different concentrations. A system where the isomerisation rate is rate limiting would result in a non-linear curve. This indicates that a prolonged isomerisation phase, as a result of lower monomeric concentrations, did not diminish the effect on association rates. Regarding the interaction between Aβ^40WT^ monomers and Aβ^40Arc^ fibrils the rate of assembly could not be monitored below 500 nM and a likely explanation is that the concentration corresponding to the KD of the interaction is reached. Although the KD value for the interaction between free Aβ^40WT^ monomer and Aβ^40Arc^ fibrils could not be determined as the signal to noise ratio was too low this a higher KD value is supported by an increased level of dissociation (Fig. 2c) suggesting looser interactions and a competing back-reaction during the isomerisation step.

A competition study was moreover carried out to investigate the different docking abilities of Aβ^40WT^ and Aβ^40Arc^ monomers on Aβ^40Arc^ fibrils (Fig. 5b). The results indicate that Aβ^40WT^ monomer docking cannot compete with Aβ^40Arc^ monomer docking onto Aβ^40Arc^ fibrils, indicating an impaired ability of Aβ^40WT^ to dock with Aβ^40Arc^ fibrils. The decay rate is however, affected suggesting that a fraction of incorporated Aβ40^WT^ significantly interferes with the overall stability of the fibril.

## Discussion

To fully understand the self-assembly of Aβ fibrils with the ultimate goal of inhibiting Aβ formation as a treatment for AD, it is important to characterise both the fibril structural architecture and the mechanisms of fibril formation. The intrinsic properties of amyloid fibrils make a detailed molecular characterisation technically challenging and the classical methods for structural elucidation, such as X-ray crystallography and solution NMR, cannot be directly applied. We have therefore developed a methodology based on quenched H/D exchange combined with NMR spectroscopy to monitor the architecture and dynamics of Aβ fibril assemblies. The method quantitatively identifies the residues involved in the fibrillar core [19], [20], [21], [22], [23]. Using solvent protection analysis, we can conclude that the fibrillar architectures of Aβ^40WT^ and Aβ^40Arc^ are strikingly similar, with only minor differences for residues at the mutation site and the C-terminal end. However, detailed analysis of the fibril formation kinetics showed that Aβ^40WT^ and Aβ^40Arc^ fibril types differ in their ability to template a polymerisation reaction.

The results presented here from cross-seeding experiments show that monomeric Aβ^40Arc^ cross-reacted easily with fibrils formed by Aβ^40WT^. This is likely a result of the higher freedom of motion due to the introduction of glycine at position 22. Aβ^40WT^ was easily incorporated within the fibrillar form of Aβ^40WT^ but not Aβ^40Arc^. Through kinetic analysis, we showed that Aβ^40WT^ seeded on Aβ^40Arc^ exhibited a higher dissociation rate, indicating a competing back-reaction where alternative conformations results in increased dissociation of the monomer from the fibril. A preceding cross-seeding event did not change the behaviour, suggesting that the effect is a consequence of the fibrillar architecture rather than the specific monomer sequence.

In the dock and lock model, the addition of a peptide onto the fibrillar end is energetically unfavourable unless the previous peptide has adopted the fibrillar conformation. This means that prolongation of the maturation step (i.e. the time between incorporation of two subsequent peptides onto the fibril) would favour an increase in the fraction of high affinity bound peptides. As a consequence, the time-dependent isomerisation event would be enhanced at lower monomer concentrations where the time between each new docking event would be longer, thereby increasing the maturation time. However, our results showed that the impaired ability of Aβ^40WT^ to bind to the fibrillar form of Aβ^40Arc^ was not compensated for by lowering the monomer concentration. This suggests that the affinity between Aβ^40WT^ monomers and the fibrillar end of Aβ^40Arc^ is impaired. A direct measurement of the association between free Aβ^40WT^ monomers and the fibrillar ends of Aβ^40Arc^ was not possible due to a poor signal to noise ratio. The significantly higher dissociation rate noted upon probing Aβ^40^ on Aβ^40Arc^ fibrils, however, suggests a competing back-reaction and also a lower binding strength affinity. Competition studies between monomeric Aβ^40WT^ and Aβ^40Arc^ were performed as an alternative approach to evaluate the ability of Aβ^40WT^ to bind to the fibrillar form of Aβ^40Arc^. The results showed that Aβ^40WT^ monomers are essentially unable to interfere with binding of Aβ^40Arc^ to Aβ^40Arc^ fibrils. However, Aβ^40WT^ monomers affected the dissociation rate. This could possibly be the consequence of a small fraction of incorporated Aβ^40WT^ monomers introducing weak links in the Aβ^40Arc^ fibrils. This is consistent with a previous report where mixtures of Aβ40^Arc^ and Aβ^40WT^ stabilised the oligomeric state of Aβ^40Arc^ and thereby prolonged its maturation into amyloid fibrils [30].

From our results, we can conclude that incorporation of Aβ^40WT^ monomers into the fibrillar form of Aβ^40Arc^ is significantly impaired as result of a reduced ability to dock and isomerise relative to Aβ^40Arc^ monomers. Upon docking, the fibril structure is determined by a balance between intra-peptide and peptide-fibril interactions. At this point, it is not possible to determine if the impaired incorporation of Aβ^40WT^ to adopt into Aβ^40Arc^ fibrils is due to structural limitations of monomeric Aβ^40WT^ in solution, interactions of the monomer with the fibril end or a combination of the two effects. Nonetheless, Aβ^40WT^ monomer binding to Aβ^40Arc^ is of low affinity and cannot compete efficiently with the Aβ^40Arc^ monomer binding. Therefore, the properties of Aβ fibrils are controlled to a great extent by the properties and constraints of the precursor molecules.

From a general point of view, the initial formation of a nucleus and the architecture of the resulting amyloid fibrils are controlled by both thermodynamic and kinetic factors. As the aggregates increase in size, the energetic barriers between different states increases and kinetic barriers essentially block interconversion between different fibrillar forms. On this basis we hypothesise that the predominant fibrillar structure would be the structure with the fastest kinetics of formation even though more thermodynamically stable states might exist. Interestingly, this hypothesis is supported by the results shown here as the measurements of affinity between the monomers and the fibrillar ends are directly related to the thermodynamic stability of the fibril [27], [28]. Our results show that Aβ^40Arc^ monomers have a significantly stronger affinity for Aβ^40WT^ fibrils than Aβ^40Arc^ fibrils. Since Aβ^40Arc^ monomers can adopt the conformation of both Aβ^40WT^ and Aβ^40Arc^ fibrils, it is not thermodynamic stability that determines fibrillar architecture but rather the rate of formation. Due to the high kinetic barriers, a subsequent re-arrangement of the fibril into a thermodynamic more stable form is prevented.

Development of AD is the consequence of an imbalance between aggregate formation and degradation. The rate of peptide assembly as well as the stability of the formed aggregates is consequently of high importance. We can within this work show that the solvent protection patterns of the fibrillar forms of Aβ^40WT^ and Aβ^40Arc^ are similar and suggest a similar structure overall. However, through cross-seeding experiments, striking differences regarding aggregation rates was seen, indicating structural differences. Our results suggest that Aβ^40WT^ docking and subsequent isomerisation into Aβ^40Arc^ fibrils is restricted. This finding highlights the importance of structural constraints at an early point in the process of incorporating free monomers into Aβ fibrils. As a consequence, structural constraints of the monomer, possibly already in solution, determine the rate of fibril assembly. We further showed that formation of the predominant Aβ fibrillar architecture is controlled by kinetics rather than thermodynamics where the most thermodynamically stable form is not necessarily the predominant structure within a sample.
